# Early Outcomes of the PhysioFlex Semi-Rigid Open Annuloplasty Ring: A Minimally Invasive Mitral Valve Repair Cohort Analysis

**DOI:** 10.3390/jcm14124155

**Published:** 2025-06-11

**Authors:** Martina Dini, Serdar Akansel, Kristin Wilkens, Emilija Miskinyte, Stephan Jacobs, Volkmar Falk, Jörg Kempfert, Markus Kofler

**Affiliations:** 1 Department of Cardiothoracic and Vascular Surgery, Deutsches Herzzentrum der Charité (DHZC), Augustenburger Platz 1, 13353 Berlin, Germany; 2 Charité—Universitätsmedizin Berlin, Freie Universität Berlin and Humboldt-Universität zu Berlin, Charitéplatz 1, 10117 Berlin, Germany; 3 DZHK (German Centre for Cardiovascular Research), Partner Site Berlin, 10785 Berlin, Germany; 4 Department of Health Sciences and Technology, Translational Cardiovascular Technologies, Institute of Translational Medicine, Eidgenoessische Technische Hochschule (ETH) Zurich, Rämistrasse 101, 8093 Zurich, Switzerland

**Keywords:** mitral valve repair, minimally invasive, ring annuloplasty, semi-rigid annuloplasty ring

## Abstract

**Objectives**: New generations of annuloplasty rings are designed to combine structural support with enhanced flexibility, allowing for better adaptation to the dynamic nature of the mitral annulus. This study investigated the early clinical and echocardiographic outcomes of minimally invasive mitral valve repair (MI-MVr) with a new semi-rigid open ring (PhysioFlex, annuloplasty ring, Edwards Lifesciences, Irvine, CA, USA). **Methods**: A total of 150 consecutive patients who underwent MI-MVr for severe degenerative mitral regurgitation (DMR) using the PhysioFlex annuloplasty ring between June 2021 and April 2024 were included in the study. Preoperative, intraoperative, and postoperative data were collected for the entire population. A three-dimensional mitral valve reconstruction analysis was performed across the entire cohort using a semi-automated software package (4D Mitral Valve Analysis; Tomtec Imaging Systems, Munich, Germany). **Results**: The median age was 59 (50–67) years and 25.3% patients were female. The median Euroscore II and left ventricular ejection fraction were 0.72 (0.56–0.99) and 60% (55–65), respectively. The median implanted ring size was 34 mm (32–36). The entire cohort was discharged with no residual mitral regurgitation greater than mild and a median mean transmitral pressure gradient of 2.4 mmHg (2–3). The median hospitalization time was 7 days (6–11) and in-hospital mortality occurred in 1 (0.7%) patient. **Conclusions**: MI-MVr was safely performed using the novel semi-rigid partial PhysioFlex ring in the DMR patient cohort with favorable early results. The PhysioFlex annuloplasty ring may be used as an alternative to complete rings in MI-MVr. Further research is needed to conduct comparisons with other currently available annuloplasty rings.

## 1. Introduction

Mitral valve repair (MVr) represents the treatment-of-choice in severe degenerative mitral valve regurgitation (DMR) [1], offering better long-term survival rates compared to mitral valve replacement. The preservation of native mitral valve tissue in combination with a preserved ejection fraction and low rates of recurrent mitral regurgitation are cornerstones for improved long-term survival and quality of life [2,3,4]. A minimally invasive approach was proven to be safe in terms of hard clinical endpoints and is associated with shorter hospital length of stay and lower rates of blood transfusion [5,6,7].

Ring annuloplasty in MVr is a standard technique that stabilizes the mitral annulus and enhances the durability of the repair [8,9,10]. The selection of the appropriate ring type is crucial in MVr, as it plays a key role in restoring the physiological annular shape and ensuring a durable repair by preventing the re-dilatation of the mitral annulus. It should be based on a patient’s mitral annular anatomy and mitral regurgitation (MR) etiology [8,11,12]. This study aims to evaluate the early clinical outcomes and annular dynamics of a new open, semi-rigid, saddle-shaped ring in a cohort undergoing minimally invasive mitral valve repair (MI-MVr) for severe DMR.

## 2. Materials and Methods

### 2.1. Study Design

**Figure 1:**
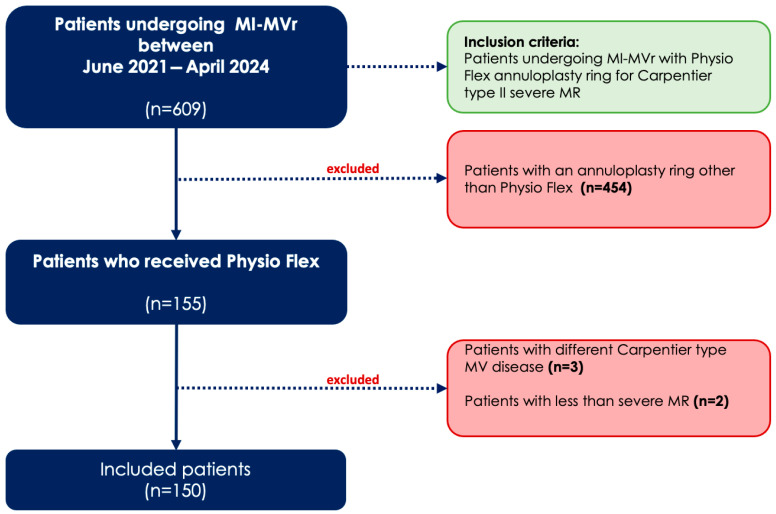
Study flowchart showing the study population and inclusion and exclusion criteria. MI-MVr = minimally invasive mitral valve repair; MR = mitral valve regurgitation; MV: mitral valve.

This study is a single-center, retrospective analysis of a patient cohort undergoing MI-MVr using the PhysioFlex annuloplasty ring (Edwards Lifescience, Irvine, CA, USA) for Carpentier type II mitral valve (MV) pathology between June 2021 and April 2024. Patients who received an annuloplasty ring other than PhysioFlex (n = 454) and those with non-degenerative MR etiologies (n = 3) were excluded from the study. A study flowchart is provided in Figure 1.

A total of 150 consecutive patients who met the inclusion criteria underwent a three-dimensional (3D) MV reconstruction analysis.

Baseline characteristics and other patient-related data were collected from electronic medical records. The institutional ethical committee approved the study for the use of patient data (Charité Ethical Committee, Berlin, approval number EA4/041/21). Individual patients’ informed consent was waived due to the retrospective design of the study.

### 2.2. Three-Dimensional Reconstruction of the Mitral Valve

**Figure 2:**
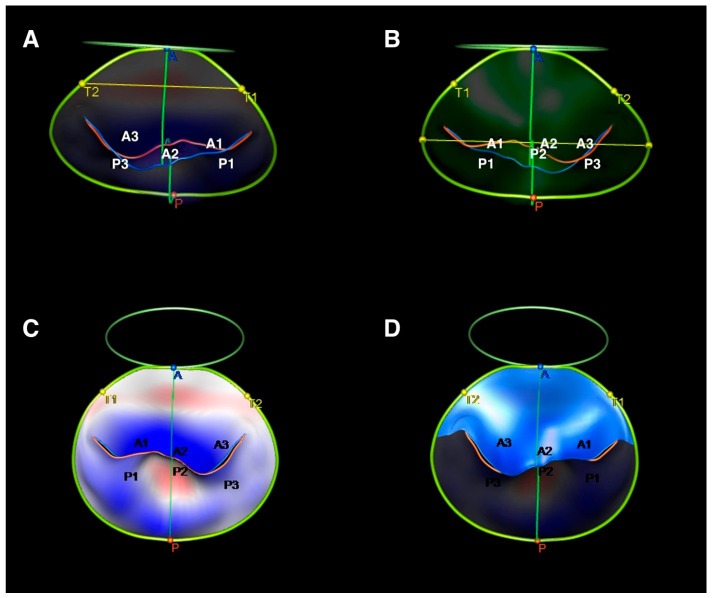
3D echocardiographic reconstruction of the mitral valve using Tomtec. (**A**) Measurement of the intertrigonal distance; (**B**) measurement of the commissural width; (**C**) measurement of the annulus area; (**D**) measurement of the anterior leaflet area.

Images were first obtained via the volumetric acquisition of the ultrasound datasets immediately after anesthesia induction and weaning from cardiopulmonary bypass. 3D transesophageal echocardiography (TEE) was performed in all patients with a commercially available ultrasound system (Vivid9; GE Vingmed Ultrasound AS, Horten, Norway). Then, these pre-MR and post-MVr 3D TEE data were analyzed using a semi-automated four-dimensional (4D) mitral valve assessment tool using Tomtec-Arena version 2.31 (Tomtec Imaging Systems, Munich, Germany), as previously described [13]. The ECG-based mid-systolic point of the cardiac cycle is automatically defined by the software. A static 3D mitral reconstruction is generated following the placement of landmarks on the mitral annulus, the coaptation point, the aortic–mitral continuity and the aortic annulus, as illustrated in Figure 2. After manual corrections of the generated model are conducted, if needed, the software creates a dynamic model that reflects the systolic motion of the MV, depending on the initial adjustment. Several MV measurements are provided after the approval of the dynamic model. All 3D-MV reconstruction analyses were performed on the mid-systolic frame pre-operatively and postoperatively by a single-experienced observer who was blinded to the operative details.

The antero-posterior (AP) diameter is defined as the distance between the anterior high point and the posterior high point of the mitral annulus and is calculated in centimeters.

The antero-lateral to postero-medial (AL-PM) diameter is defined as the diameter between anterolateral and posteromedial landmarks and is calculated in centimeters.

The annulus circumference is defined as the total perimeter of the annular edge and is calculated in centimeters.

The annulus area is defined as the area enclosed by the 3D projection of the annulus and is calculated in squared centimeters.

The annulus height is defined as the vertical distance between highest and lowest annular points and is calculated in centimeters.

The annulus height to commissural width ratio (AHCWR) is calculated as the ratio of the mitral annular height to the commissural width. The annulus height to commissural width ratio (AHCWR) is calculated as the ratio of the mitral annular height to the commissural width. It is a measurement used to assess the shape and non-planarity of the mitral valve annulus and it represents a surrogate for the saddle shape of the mitral annulus. A preservation of the AHCWR after MVR represents a preservation of the native saddle-like shape of the MV.

### 2.3. Surgical Technique

All patients underwent preoperative CT scans to understand their cardiac and vascular anatomy and to avoid cannulation and access-related complications. Minimally invasive mitral valve surgery (MI-MVS) via a 3–4 cm right lateral or periareolar mini-thoracotomy was the surgical approach employed in all individuals, as described in previous reports [14,15]. The operations were performed with either video-assisted or 3D fully endoscopic visualization (3D high-definition endoscopic system, Aesculap AG, Tuttlingen, Germany) by the specialized MI-MVS surgical team. Cardio-pulmonary bypass was initiated via the cannulation of the femoral vein and artery using an ultrasound-guided percutaneous or cut-down technique. Systemic hypothermia to 34 °C was introduced to establish a DO_2_-guided perfusion. Cardiac arrest was induced using antegrade cold Del Nido cardioplegia solutions following cross-clamping via a transthoracic Chitwood cross-clamp or endoaortic balloon occlusion system (IntraClude System, Edwards Lifesciences, Irvine, CA, USA), based on the surgeons’ preferences and patients’ anatomy, as previously described [16].

### 2.4. Statistical Analysis

Descriptive data are provided as the median and interquartile range (IQR) for asymmetrically distributed continuous variables and as the mean and standard deviation for symmetrically distributed continuous variables. Categorical variables are presented as counts and percentages. Analyses were performed using R software version 4.3.3 (R Foundation for Statistical Computing: Vienna, Austria) [17]. A dependent paired *t* test was used to compare the preoperative and postoperative echocardiographic parameters obtained from the four-dimensional mitral valve assessment; *p* < 0.05 was considered statistically significant. All statistical data were presented in accordance with the statistical and data reporting guidelines of the European Journal of Cardio-Thoracic Surgery and the Strengthening the Reporting of Observational Studies in Epidemiology (STROBE) statement on guidelines for observational studies [18,19].

### 2.5. Data Availability Statement

The data obtained for this research cannot be shared publicly due to data protection policies but will be shared with interested parties upon reasonable request to the corresponding author.

## 3. Results

### 3.1. Patient Characteristics

The median age was 59 (50–67) years, and 38 patients (25.3%) were female. The median body mass index and median body surface area were 24.50 (22.40–26.57) Kg/m^2^ and 1.98 (1.82–2.09) m^2^, respectively. The median EuroSCORE II and median STS score were 0.361% (0.234–0.598) and 0.361% (0.234–0.598), respectively, as shown in Table 1.

In total, 45 patients (30%) were in New York Heart Association functional class III and 5 patients (3.3%) were in class IV. Most operations were performed electively, but four patients (2.7%) were admitted in a critical state, four patients (2.7%) underwent urgent interventions, and one patient (0.7%) underwent an emergency intervention.

All patients presented with severe Carpentier type II MR. The median left ventricle ejection fraction (LVEF) was 60 (55–65) %, and the median left ventricle end diastolic diameter (LVEDD) was 58 (53–62) mm. Anterior leaflet disease was observed in 17 patients (11.3%). Among them, 14 patients (9.3%) had a prolapse, 2 patients (1.3%) had flail, 1 patient (0.7%) had billowing, and 1 patient (0.7%) had a cleft in addition to a prolapse.

Posterior mitral leaflet disease was identified in 138 patients (92%), with prolapse observed in 97 patients (64.7%), flail in 41 patients (27.3%), and cleft in 50 patients (33.3%) in addition to prolapse. Bileaflet disease was observed in 11 patients (7.3%) in the cohort. Complete details regarding patients’ preoperative characteristics are listed in Table 1.

### 3.2. Mitral Valve Repair

Cardiopulmonary bypass was established via the percutaneous cannulation of the femoral artery and vein in 102 patients (68%) and using the cut-down technique in the remaining cases (n = 48, 32%). Most patients (n = 145, 96.7%) underwent MI-MVr with 3D fully endoscopic visualization. MI-MVr was performed via a 3–4 cm right lateral minithoracotomy technique in 125 patients (83.3%) and a periareolar “nipple cut” incision in 25 patients (16.7%), based on the surgeon’s choice and patient’s anatomy. Aortic cross-clamping was performed with an endoaortic balloon occlusion system in 74 patients (49.3%) and via transthoracic Chitwood aortic cross-clamp in 76 patients (50.7%). A PhysioFlex annuloplasty ring was successfully implanted in all patients, and the median implanted ring size was 34 (32–36) mm. There was no conversion to sternotomy. Complete details of intraoperative data are given in Table 2.

### 3.3. Postoperative Course

The postoperative course is summarized in Table 3.

The median length of hospital stay was 7 (6–11) days and median intensive care unit stay was 23 (18–30) h. The median duration of mechanical ventilation was 6.7 (5.1–10.6) h.

In the discharge echocardiographic evaluation, the median LVEF was 50 (44–55)%, and the median LVEDD was 52 (48–56.25) mm. The mean transmitral pressure gradient was 2.4 (2–3) mmHg. There was no patient with greater-than-mild MR at discharge. Perioperative and discharge echocardiography revealed no signs of systolic anterior movement (SAM).

Prolonged mechanical ventilation was observed in eight patients (5.3%).

Postoperative acute renal failure was observed in one patient (0.7%), who required dialysis.

**Figure 3:**
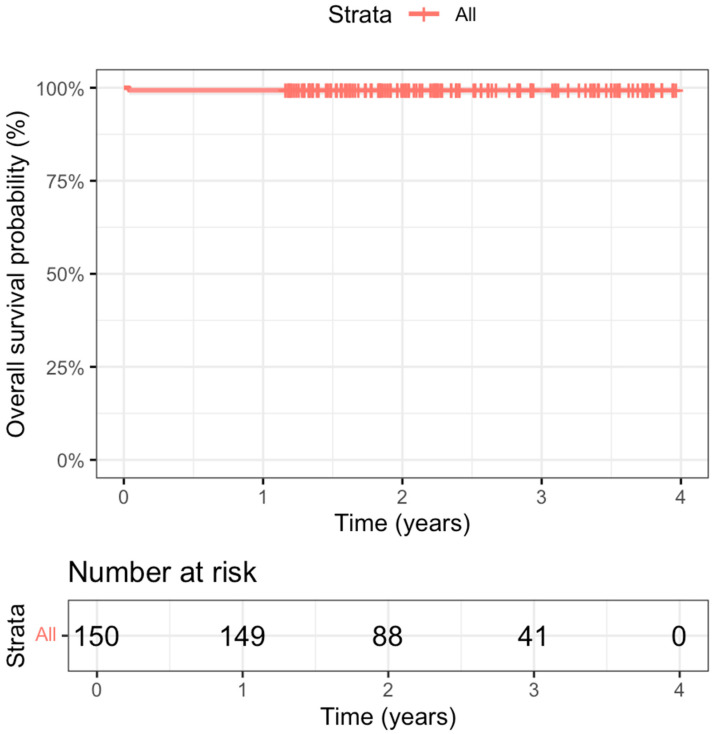
Kaplan–Meier curve survival analysis.

Iatrogenic occlusion of the circumflex coronary artery occurred in one patient (0.7%) and was managed using a combined surgical and percutaneous approach. The annular sutures causing the kinking of the LCx were repositioned; this was detected using a real-time coronary angiogram, as reported previously [20]. In-hospital mortality was observed in one patient (0.7%) due to respiratory insufficiency and multiorgan failure on the 14th postoperative day. At the one-year follow-up examination, there was no additional mortality. Thus, the overall survival rate was 99.33% at the one-year follow-up, as represented in the Kaplan–Meier curve shown in Figure 3.

### 3.4. Mitral Annular Parameters

Mitral annular parameters obtained using a 4D-MV assessment tool are presented in Table 4.

**Figure 4:**
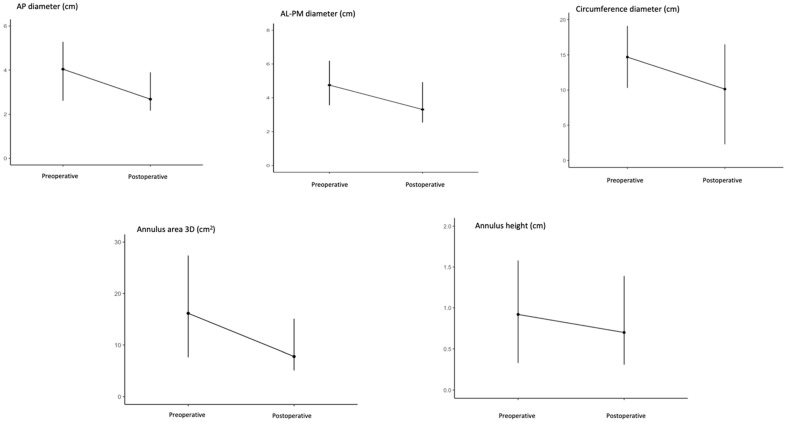
Changes in preoperative and postoperative mitral annular parameters. Error bars showing the decrease in mitral annular parameters from preoperative to postoperative evaluations. AL-PM: anterolateral to posteromedial; AP: antero-posterior; 3D: three dimensional.

AP and AL-PM diameters demonstrated reductions of 31.9% and 30.4%, with mean differences of 1.35 cm (*p* < 0.001) and 1.48 cm (*p* < 0.001), respectively. Similarly, the annulus circumference decreased by 30.9%, with a mean difference of 4.78 cm (*p* < 0.001). A graphical representation of the reduction in all parameters is given in Figure 4. A more pronounced reduction (51.7%) was observed in the 3D annulus area, with a mean difference of 8.68 cm^2^ (*p* < 0.001). The annulus height significantly decreased after MVr (24.5%, with a mean difference of 0.25; *p* < 0.001). The annulus height to AHCWR was preserved after MVr, with a mean difference of 0.01 (*p* = 0.21).

## 4. Discussion

In the present study, we aimed to report our experience using a recently introduced semi-rigid open ring (PhysioFlex) in a minimally-invasive mitral valve repair cohort.

The main findings of the study can be summarized as follows; (1) after MVr, the AP and AL-PM diameters, as well as the annulus area, decreased significantly while AHCWR was preserved; (2) there was no patient with greater-than-mild MR or SAM according to the discharge echocardiography; (3) 30-day mortality was observed in one patient; (4) all patients were discharged with low mean transmitral pressure gradients.

The currently available annuloplasty rings differ in terms of consistency, conformation, and shape. The choice of the ring should be based on the patient’s characteristics, with regards to mitral valve anatomy and disease, which represent an important determinant of mitral annulus changes after repair.

As for patients with reduced left ventricular function, the choice of a rigid ring is preferable to support reverse remodeling. In Carpentier type II MV disease, excessive leaflet motion is observed due to elongated or ruptured chordae [21]. In this case, a marked remodeling is usually not necessary and semi-rigid or flexible rings are preferable.

Despite their marked remodeling capabilities, rigid rings create a rigid planar annulus area with the consequent limitation of physiological dynamics and annular saddle shaping throughout the cardiac cycle [8]. Restricted annular mobility during the cardiac cycle contributes to the obstruction of the left ventricular outflow tract [22,23], extending to aortic curtain mobility [24]. These findings have been confirmed by Veronesi et al. [25], who showed impaired aorto-mitral coupling and reduced aortic annular motion in axial and longitudinal plane after a complete ring annuloplasty. Semi-rigid annuloplasty rings offer flexibility to accommodate annulus changes during the cardiac cycle [26] while maintaining the necessary rigidity to allow for annular remodeling [27].

Closed rings are associated with a higher incidence of SAM, particularly in cases of excessive annular downsizing and an excess of leaflet tissue in narrow left ventricular outflow tracts [28,29,30]. Closed conformation of the ring also plays a role in impairing the aorto-mitral curtain’s function by reducing the outflow diameter and limiting the anterior mitral leaflet’s movement. It has been reported that a complete ring annuloplasty immobilizes the aortomitral curtain and impairs the filling and emptying mechanisms of the left ventricle by reducing the left ventricular outflow tract’s diameter and anterior mitral leaflet motion [31].

PhysioFlex is one of the latest generations of semi-rigid, open, saddle-shape rings with an in-plane progressive flexibility. The flexibility of the PhysioFlex ring provides more physiological support by preserving valvular native geometry compared to other available semi-rigid rings. Its open conformation presents an asymmetrical anterior open segment and a complete posterior segment, as well as a progressive saddle height, that optimizes chordal force distribution and reduces leaflet and chordal stress [32,33].

In patients with Carpentier type II MV disease, PhysioFlex should provide remodeling due to its semi-rigid conformation, while preserving the physiological motion of the anterior mitral annulus, by virtue of its saddle-like shape and flexibility. The open ring conformation should also provide support to the posterior mitral annulus at the same time as preventing SAM [28] and maintaining aorto-mitral coupling dynamics.

The early clinical outcomes of MVr using various annuloplasty rings show comparable data to those reported in our study. Noack et al. [34] reported a mild residual MR at discharge in 18% of patients and a rate of moderate residual MR at discharge of 1.6%, with a mean transmitral pressure gradient of 2.6 (1.1) mmHg when using a semi-rigid saddle-shaped closed ring. Vohra et al. [35] reported an in-hospital mortality rate of 1%, mild residual MR of 13%, and a mean transmitral pressure gradient of 3.5 (1.2) mmHg with the same ring. Bruno et al. [26] reported 3.3% in-hospital death, a mild residual MR of 17.4%, and a mean transmitral pressure gradient of 3.3 (1.5) mmHg when using a semi-rigid ring. Fiore et al. [36] reported a mild residual MR of 5.7%, moderate residual MR of 1.9%, and a mean transmitral gradient of 3.3 (1.2) mmHg when using a semi-rigid ring.

Our findings of reduced AP, AL-PM diameter, and mitral annulus area align with the findings of Mahmood et al. [37], who observed similar reductions in annular geometry in two cohorts of patients receiving either a saddle-shaped ring or a flat ring.

In our study, PhysioFlex implantation led to favorable early clinical outcomes with a good control on MR, as well as the absence of SAM and left ventricle outflow tract obstruction, both in postoperative assessments and at the discharge echocardiographic evaluation.

We acknowledge that this study has several limitations. First, its retrospective single-center, non-comparative study design may limit the power of the findings. Second, as this study was performed in a single high-volume center, the results may not be generalizable to lower-volume institutions or less experienced surgeons. The findings are based on early postoperative data and should be confirmed through future studies with mid- and long-term echocardiographic and clinical data. Therefore, future prospective studies are needed to address these limitations. Finally, it should be noted that multiple co-authors of the present study have financial links with the manufacturer of the ring (i.e., educational grants including travel support, fees for lectures and speeches, fees for professional consultation, and research and study funds).

## 5. Conclusions

The PhysioFlex annuloplasty ring is associated with favorable short-term hemodynamic and echocardiographic results in terms of the mean postoperative mitral pressure gradient, residual MR, and SAM occurrence. Further studies that evaluate mid- and long-term MVr durability are necessary to confirm these preliminary results, ideally in a comparative setting with other currently available annuloplasty rings.

## Figures and Tables

**Table 1:** Baseline characteristics.

N (%)/Median [IQR]	n = 150
Age (years)	59 (50–67)
Gender	
Female	38 (25.3)
BMI (Kg/m^2^)	24.50 (22.40–26.57)
BSA (m^2^)	1.98 (1.82–2.09)
Hypertension	72 (48)
Dyslipidemia	42 (28)
Diabetes	9 (6)
Diet	2 (1.3)
Oral therapy	5 (3.3)
Insulin	2 (1.3)
TIA/Stroke	4 (2.7)
Status post myocardial infarction	3 (2)
Peripheral artery disease	2 (1.3)
Chronic lung disease	9 (6)
Atrial fibrillation	33 (22)
Paroxysmal	22 (14.7)
Persistent	8 (5.3)
Permanent	3 (2)
Chronic kidney disease	10 (6.7)
EuroSCORE II (%)	0.72 (0.56–0.99)
STS Score (%)	0.361 (0.234–0.598)
Preoperative NYHA class	
I	35 (23.3)
II	65 (43.3)
III	45 (30)
IV	5 (3.3)
Preoperative critical state	4 (2.7)
Active endocarditis	1 (0.7)
Urgency of intervention	
Urgency	4 (2.7)
Emergency	1 (0.7)
LVEF (%)	60 (55–65)
LVEDD (mm)	58 (53–62)
Right ventricular systolic pressure (mmHg)	35.95 (28.45–50)
Pulmonary hypertension	
Moderate (31–55 mmHg)	30 (20)
Severe (>55 mmHg)	13 (8.7)
Mitral annular calcification	3 (2)
Anterior mitral leaflet disease	
Prolapse	14 (9.3)
Flail	2 (1.3)
Billowing	1 (0.7)
Cleft	1 (0.7)
Posterior mitral leaflet disease	
Prolapse	97 (64.7)
Flail	41 (27.3)
Cleft	50 (33.3)

Data are presented as the number (percentages) and median (interquartile range). BMI: body mass index; BSA: body surface area; IQR: interquartile range; LVEDD: left ventricle end diastolic diameter; LVEF: left ventricle ejection fraction; NYHA: New York Heart Association; STS: Society of Thoracic Surgeons; TIA: transient ischemic attack.

**Table 2:** Intraoperative characteristics.

N (%)/Median (IQR)	n = 150
Operation time (min)	157 (141–187.5)
Cardio-pulmonary bypass time (min)	107 (90.25–127.75)
Cross-clamp time (min)	63.50 (56–85)
Nipple cut	25 (16.7)
Type of femoral cannulation	
Percutaneous	102 (68)
Open	48 (32)
3D endoscopic system	145 (96.7)
Type of aortic cross-clamp	
Endoaortic balloon occlusion system	74 (49.3)
Transthoracic Chitwood cross-clamping	76 (50.7)
Concomitant procedure	
Tricuspid valve repair	3 (2)
Maze/LAAC	28 (18.7)
ASD Closure	7 (4.7)
Ring size (mm)	34 (32–36)

Data are presented as the number (percentages) and median (interquartile range). ASD: atrial septum defect; IQR: interquartile range; LAAC: left atrial appendix closure; 3D: three-dimensional.

**Table 3:** Postoperative characteristics.

N (%)/Median [IQR]	n = 150
Hospitalization time (days)	7 (6–11)
ICU stay (h)	23 (18–30)
Ventilation time (h)	6.7 (5.1–10.6)
LVEF (%)	50 (44–55)
LVEDD (mm)	52 (48–56.25)
Right ventricular systolic pressure (mmHg)	25.40 (22.75–30)
Transmitral mean pressure gradient	2.4 (2–3)
Residual MR at discharge	
Mild	23 (15.3)
Moderate	0 (0)
Severe	0 (0)
Revision for bleeding	7 (4.7)
Myocardial infarction	1 (0.7)
Stroke	2 (1.3)
New onset of atrial fibrillation	20 (13.3)
Respiratory complications	14 (9.3)
Respiratory insufficiency	5 (3.3)
Respiratory failure	3 (2)
Renal complications	
Renal insufficiency	3 (2)
Renal failure (dialysis)	1 (0.7)
Gastrointestinal complications	1 (0.7)
In-Hospital death	1 (0.7)

Data are presented as the number (percentages) and median (interquartile range). ICU: intensive care unit; IQR: interquartile range; LVEDD: left ventricle end diastolic diameter; LVEF: left ventricular ejection fraction; MR: mitral regurgitation.

**Table 4:** Mitral annulus parameters.

Mean (SD)	Preoperative Assessment	Postoperative Assessment	Percentage of Reduction	Mean Difference	*p* Value
AP diameter (cm)	4.05 (0.55)	2.76 (0.38)	31.9	1.35	<0.001
AL-PM diameter (cm)	4.75 (0.61)	3.31 (0.39)	30.4	1.48	<0.001
Annulus circumference (cm)	14.69 (1.76)	10.14 (1.64)	30.9	4.78	<0.001
Annulus area 3D (cm^2^)	16.15 (3.84)	7.79 (1.86)	51.7	8.68	<0.001
Annulus height (cm)	0.92 (0.23)	0.70 (0.23)	24.5	0.25	<0.001
AHCWR	0.196 (0.44)	0.212 (0.06)	−8.6	0.01	0.21

Data are presented as the mean (standard deviation). AHCWR: annulus height to commissural width ratio; AL: anterolateral; AP: antero-posterior; PM: posteromedial; SD: standard deviation, 3D: three dimensional.

## Data Availability

The original contributions presented in the study are included in the article, further inquiries can be directed to the corresponding authors.

## Abbreviations

AHCWR	Annulus height to commissural width ratio
AL-PM	Anterolateral to posteromedial
AP	Antero-posterior
DMR	Degenerative Mitral Regurgitation
IQR	Interquartile range
LVEF	Left ventricle ejection fraction
LVEDD	Left ventricle end diastolic diameter
MI-MVr	Minimally invasive mitral valve repair
MI-MVS	Minimally invasive mitral valve surgery
MR	Mitral valve regurgitation
V	Mitral valve
MVr	Mitral valve repair
SAM	Systolic anterior movement
TEE	Transesophageal echocardiography
3D	Three dimensional
4D	Four dimensional
